# Increased drought and extreme events over continental United States under high emissions scenario

**DOI:** 10.1038/s41598-023-48650-z

**Published:** 2023-12-06

**Authors:** Sagar Gautam, Umakant Mishra, Corinne D. Scown, Rajan Ghimire

**Affiliations:** 1 Bioscience Division, Sandia National Laboratory, Livermore, CA 94550 USA; 2 Joint BioEnergy Institute, Lawrence Berkeley National Laboratory, Emeryville, CA 94608 USA; 3 Energy Analysis and Environmental Impact Division, Lawrence Berkeley National Laboratory, Berkeley, CA 94720 USA; 4 Biological Systems and Engineering Division, Lawrence Berkeley National Laboratory, Berkeley, CA 94720 USA; 5 Energy and Biosciences Institute, University of California, Berkeley, CA 94720 USA; 6 Agricultural Science Center, New Mexico State University, Las Cruces, NM 88003 USA

**Keywords:** Environmental impact, Climate and Earth system modelling, Climate-change impacts, Projection and prediction

## Abstract

The frequency, severity, and extent of climate extremes in future will have an impact on human well-being, ecosystems, and the effectiveness of emissions mitigation and carbon sequestration strategies. The specific objectives of this study were to downscale climate data for US weather stations and analyze future trends in meteorological drought and temperature extremes over continental United States (CONUS). We used data from 4161 weather stations across the CONUS to downscale future precipitation projections from three Earth System Models (ESMs) participating in the Coupled Model Intercomparison Project Phase Six (CMIP6), specifically for the high emission scenario SSP5 8.5. Comparing historic observations with climate model projections revealed a significant bias in total annual precipitation days and total precipitation amounts. The average number of annual precipitation days across CONUS was projected to be 205 ± 26, 184 ± 33, and 181 ± 25 days in the BCC, CanESM, and UKESM models, respectively, compared to 91 ± 24 days in the observed data. Analyzing the duration of drought periods in different ecoregions of CONUS showed an increase in the number of drought months in the future (2023–2052) compared to the historical period (1989–2018). The analysis of precipitation and temperature changes in various ecoregions of CONUS revealed an increased frequency of droughts in the future, along with longer durations of warm spells. Eastern temperate forests and the Great Plains, which encompass the majority of CONUS agricultural lands, are projected to experience higher drought counts in the future. Drought projections show an increasing trend in future drought occurrences due to rising temperatures and changes in precipitation patterns. Our high-resolution climate projections can inform policy makers about the hotspots and their anticipated future trajectories.

## Introduction

Global atmospheric concentrations of greenhouse gases (GHG) have significantly increased and far exceeded the pre-industrial level. These changes can be attributed to the increased use of fossil fuels and agricultural emissions^1, 2^. The rise in GHG concentrations is altering the radiative balance of the Earth's atmosphere, resulting in temperature and precipitation changes that have adverse effects on ecosystems and human well-being^3^. To understand the potential future climate scenarios, Earth System Models (ESMs) are used to simulate the trajectory of temperature and precipitation under different levels of radiative forcing, based on various possible development pathways^4–6^. According to ESM projections, unless there are substantial reductions in emissions, we can expect an increase in extreme weather events^7, 8^. Among these events, drought stands out as one of the most costly and devastating due to its far-reaching impacts across multiple sectors, including agriculture^7, 9, 10^.

ESMs play a critical role in projecting long-term climate change as they provide valuable insights into the dynamics and evolution of the climate system over extended time scales. However, these models may not be suitable for site-specific applications, particularly at the field scale, because they generate outputs at a coarse resolution (~ 100 km) and exhibit biases compared to observational data^11^. One area where biases are particularly prevalent is precipitation, where climate models often overestimate the frequency of rainy days and underestimate rainfall extremes or fluctuations in seasonal rainfall and temperatures^12^. While ESMs have improved in simulating large-scale atmospheric circulation, they lack representation of processes at the local scale^13^. A common issue with ESM outputs is the precipitation bias in daily simulations. In these simulations, precipitation events occur more frequently (drizzle bias) but with lower intensity (muted extreme) compared to observations^14, 15^ This bias has significant implications for future agricultural yield projections, as less frequent, heavy rainfall leads to increased runoff and reduced soil moisture accumulation compared to more frequent, lighter rainfall. Consequently, future projections from ESMs require bias correction or downscaling to address the biases associated with high spatial resolution. This correction process is necessary to make ESM projections more plausible for impact assessment studies and to enhance our confidence in future projections.

Drought is a period of prolonged periods of abnormally low precipitation and its severity and occurrence depends on variety of factors including soil, plant and topography^16^. Precipitation, soil moisture and streamflow are the variable which are commonly used to quantify drought^17^. Observation-based drought monitoring in the United States has revealed an increased frequency of drought events^18^. The global study based on climate model and observations has indicated an increased likelihood of the flash droughts across major global croplands^19^. Another study has also indicated increase in global flash drought occurrences, with highest increase in Europe and North America under extreme emission scenarios. These increase in drought occurrence are associated to greater evapotranspiration and precipitation deficits caused by anthropogenic climate change^20^. This type of monitoring is valuable for the development of adaptation strategies that are applicable at local and regional scales. Policymakers and stakeholders require more detailed projections encompassing the spatial and temporal distribution of future drought, extreme precipitation events, and extreme temperatures. Additionally, it is important to note that drought primarily affects agriculture, as it refers to conditions in which plants experience specific levels of moisture stress, impacting both vegetative growth and crop yield^21^. Translating climate projections into drought indices provides valuable insights into the severity and frequency of future extreme events, particularly in terms of their impact on agricultural systems. This information can assist decision-makers in formulating and implementing adaptation plans. By combining downscaled data with ground-truthing from observations, the accuracy of ESMs in projecting drought on a finer scale can be improved, enhancing their utility in assessing and predicting drought events. This study uses precipitation alone for drought projection as future datasets are readily available from ESMs, use of additional variable can help explore other land processes and its impact on drought.

The currently available downscaled climate data from CMIP5 and CMIP6, with a spatial resolution of 12 to 25 km^2^, are relatively coarse for site-specific applications^22, 23^. To address this limitation, we propose downscaling the climate data to weather stations across the continental US, making it more suitable for point/field-scale applications. In this study, we employed a statistical downscaling approach to correct the existing bias in precipitation projections from ESMs. This approach involved two steps: (1) converting data values to probabilities based on the cumulative distribution function of weather station data and climate model grid simulations, and (2) mapping the distribution of historical weather station observations and future grid-scale projections to bias-correct the projections^24, 25^. The statistical downscaling approach utilized in this study was modified to eliminate the drizzle bias observed in climate model simulations^15^. Additionally, we used the Standardized Precipitation Index (SPI) to quantify future droughts using the downscaled precipitation data. The calculation of the Standardized Precipitation Index (SPI) evaluates droughts by analyzing the likelihood of precipitation occurrences, yielding standardized values wherein zero signifies the median condition. Negative values signal drought periods, while positive values indicate wet periods^26^. Through the process of standardizing precipitation, we are able to gauge the intensity and occurrence frequency of droughts across various time scales, frequencies, and durations^24^. Moreover, apart from assessing dry periods, the drought indices also encompass calculations for wet periods, which are pivotal in quantifying future flood risks^27^. Projection of drought and calculation of extreme indices based on current and future climate variables (P and T) can provide valuable insights into changing trends and serve as crucial inputs for informing policy decisions. The Expert Team on Climate Change Detection and Indices (ETCCDI) has established a comprehensive set of indices for analyzing extremes related to precipitation (P) and temperature (T) events^28^. These indices have gained broad acceptance and are extensively utilized to assess extremes in both current and projected future climates^17, 29, 30^. In this study, in addition to drought indices, we have also incorporated ETCCDI-based extreme indices. The downscaled future trajectories of precipitation and temperature projected by ESMs can be utilized to assess and project future climate extremes, including precipitation and temperature extremes. The bias-corrected precipitation data can be employed to estimate future drought conditions, which are crucial for policymaking and developing mitigation strategies.

Previous studies have examined the potential future changes in drought frequency and severity at national, regional, and global scales^31–39^. However, these studies were conducted at coarse spatial scales and did not downscale the projected climate data to the level of weather stations. Most of the previous drought projection studies used CMIP5 ESM products^36, 40, 41^, which are known to exhibit drizzle biases as highlighted by previous studies^15, 17, 42^. Few studies have also assessed future extremes using CMIP6 climate model output, including precipitation, temperature, soil moisture, and runoff^43^. A recent study conducted in China systematically evaluated drought changes using downscaled data and found reduced uncertainty in drought projections when using site-specific downscaled data^44^. In this study, we downscaled the data from CMIP6 ESMs to weather stations and used the downscaled data to analyze the future trajectory of climate extreme events. The specific objectives of this study were to: (a) quantify the bias in CMIP6 precipitation data and create a downscaled database of CMIP6 projections for the continental United States (CONUS), and (b) project the future trajectory of drought and temperature extremes using drought and extreme indices.

## Results and discussions

### Climate model precipitation bias

**Figure 1:**
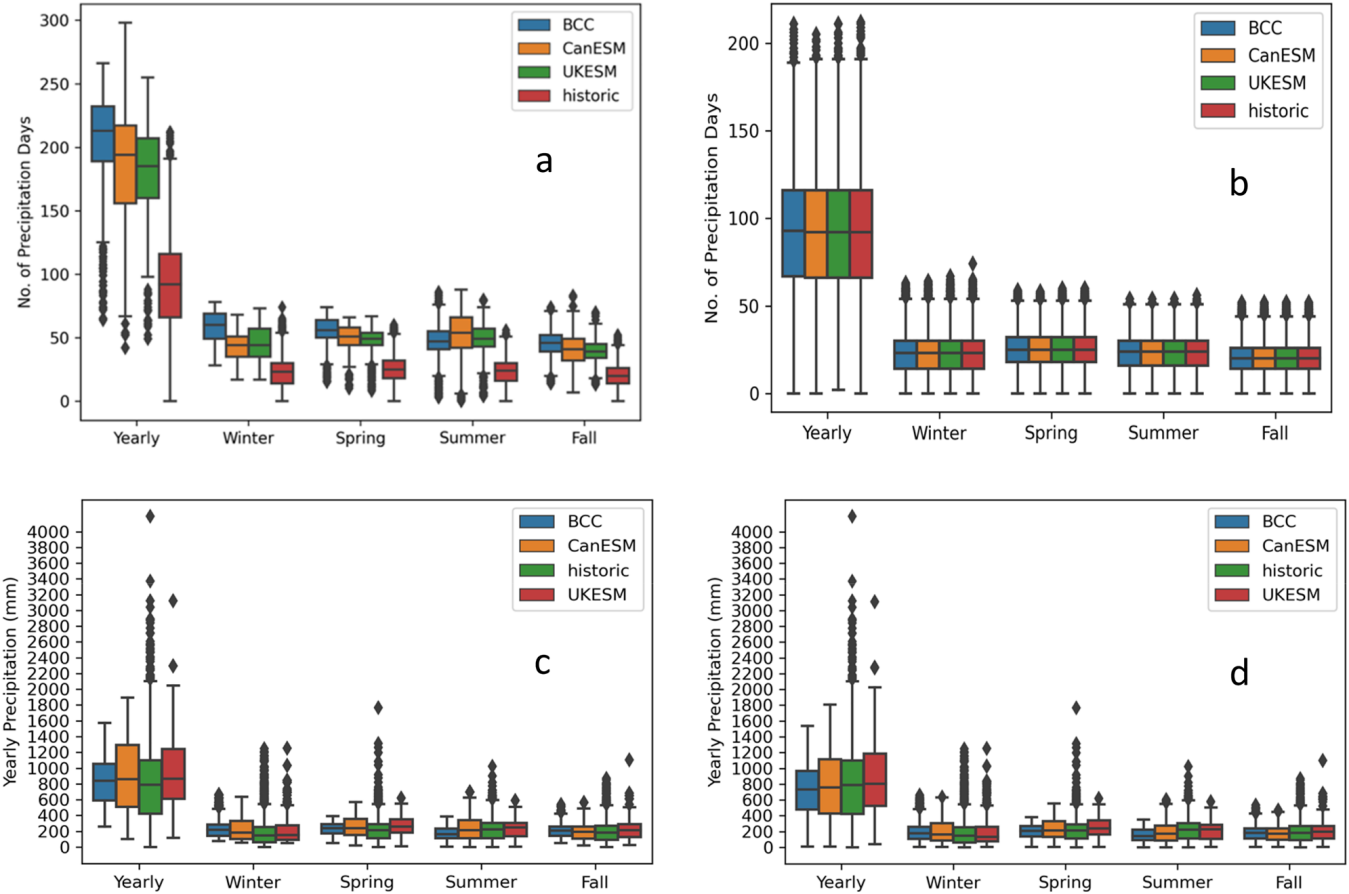
Comparison of number of precipitation days across uncorrected (**a**) corrected model (**b**) and total precipitation across uncorrected (**c**) and total precipitation across bias-corrected model (**d**). (Beijing Climate Center Climate System Model (BCC-CSM); Canadian Centre for Climate Modelling and Analysis (CanESM) and Met Office Hadley Centre (UKESM) outputs in different season with the observation during historic period (1989–2018).

Comparison of the historic observations and CMIP6 climate model projections for high emission scenarios (SSP5 8.5) showed significant bias in yearly precipitation days and total precipitation amount. The results are presented as average bounded by positive and negative values in the upper- and lowercases represent uncertainty ranges based on an interquartile range. The spatial mean annual precipitation day across CONUS was ${205}_{-17}^{+26}$, ${184}_{-29}^{+33}$ and ${181}_{-21}^{+25}$ days in BCC, CanESM and UKESM models respectively, compared to ${91}_{-25}^{+24}$ days in observations (Fig. 1a). The annual precipitation totals were ${831}_{-240}^{+223}$, ${896}_{-390}^{+397}$ and ${913}_{-303}^{+329}$ mm in BCC, CanESM and UKESM models respectively, compared to ${791}_{-971}^{+308}$ mm in observations (Fig. 1b). Similar biases in the total precipitation and number of precipitation days (wet days) were found across different seasons (Fig. 1a,b). These biases in the historical simulations were corrected using statistical downscaling. The comparison of the downscaled data with observations are shown in Fig. 1c,d. The average numbers of precipitation days and their distribution across different seasons are appropriately matched, removing the drizzle bias in the model projections (Fig. 1c). A previous regional watershed scale study for the midwestern US reported similar bias in precipitation projections in the CMIP5-ESM outputs^17^. The bias in total precipitation days was reported earlier for both CMIP3 and CMIP5 models; the bias was mostly on a large number of drizzle precipitation days and an underestimation of heavy precipitation days for different regions^15, 45^. The bias is attributed to the spatial averaging across the large spatial grids with multiple weather stations within each grid resulting in increased probability or frequency of precipitation^46, 47^. Analysis of CONUS using three different climate models with different spatial resolutions shows improvement in all aspects of simulated precipitation including spatial pattern, intensity and seasonality with the increased resolution of the models^48^. Such studies are limited to a few models, mostly covering short temporal scales, as most of the computational resources are sourced for comprehensive physical representation and increasing size of ensembles^49^. Downscaling climate data to local weather stations, as done in this study, will represent local scale variation. Downscaling the ESMs data are important before using these data in daily time scale models because the bias in ESM projections results in higher uncertainty in impact assessment studies^50^.

### Historic and future drought projections for Continental US

**Figure 2:**
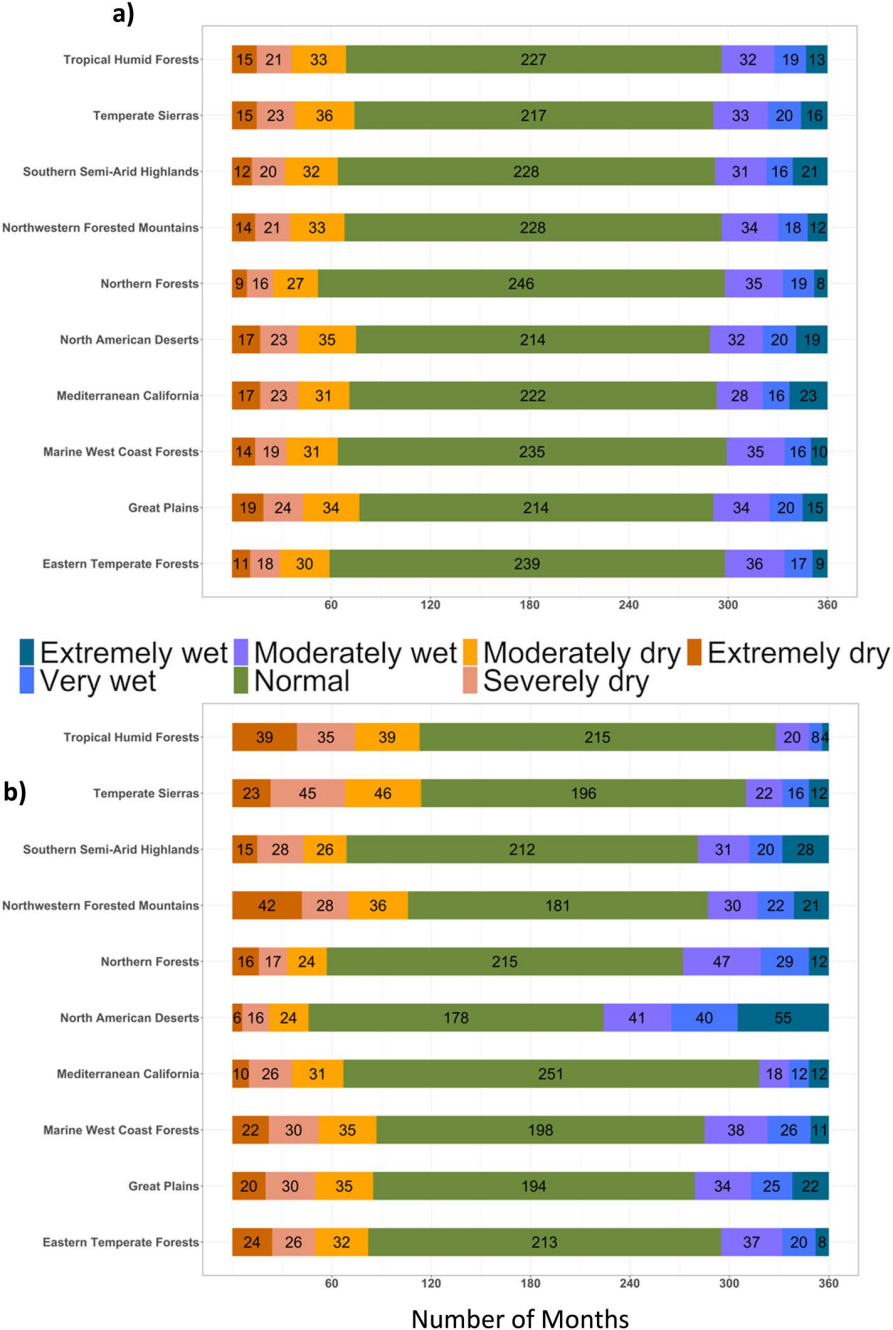
Comparison of monthly standardized precipitation index across different ecoregions over 30-year period (**a**) 30-year (1989–2018) observed period and (**b**) 30-year (2023–2052) future period based on ensemble of three ESMs. Y-axis represent different ecoregion and colors in bar represent count of each drought class.

**Figure 3:**
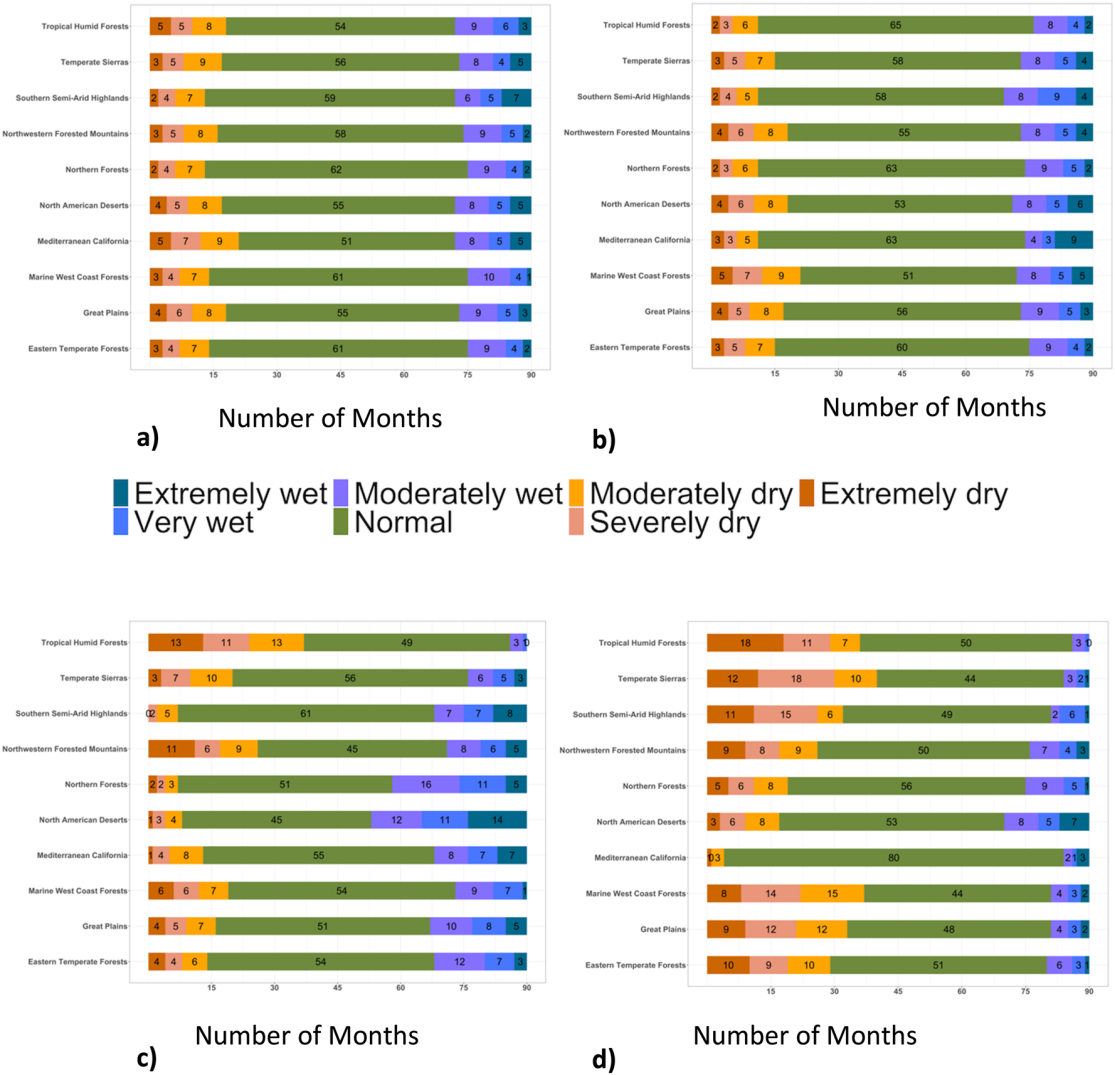
Comparison of monthly standardized precipitation index across different ecoregions for 30-year (1989–2018) observed period and 30-year (2023–2052) future period for different seasons. Upper two Figures for historic spring (**a**) and summer (**b**), and lower two figures for future spring (**c**) and summer (**d**). Y-axis represent different ecoregion and colors in bar represent count of each drought class.

**Figure 4:**
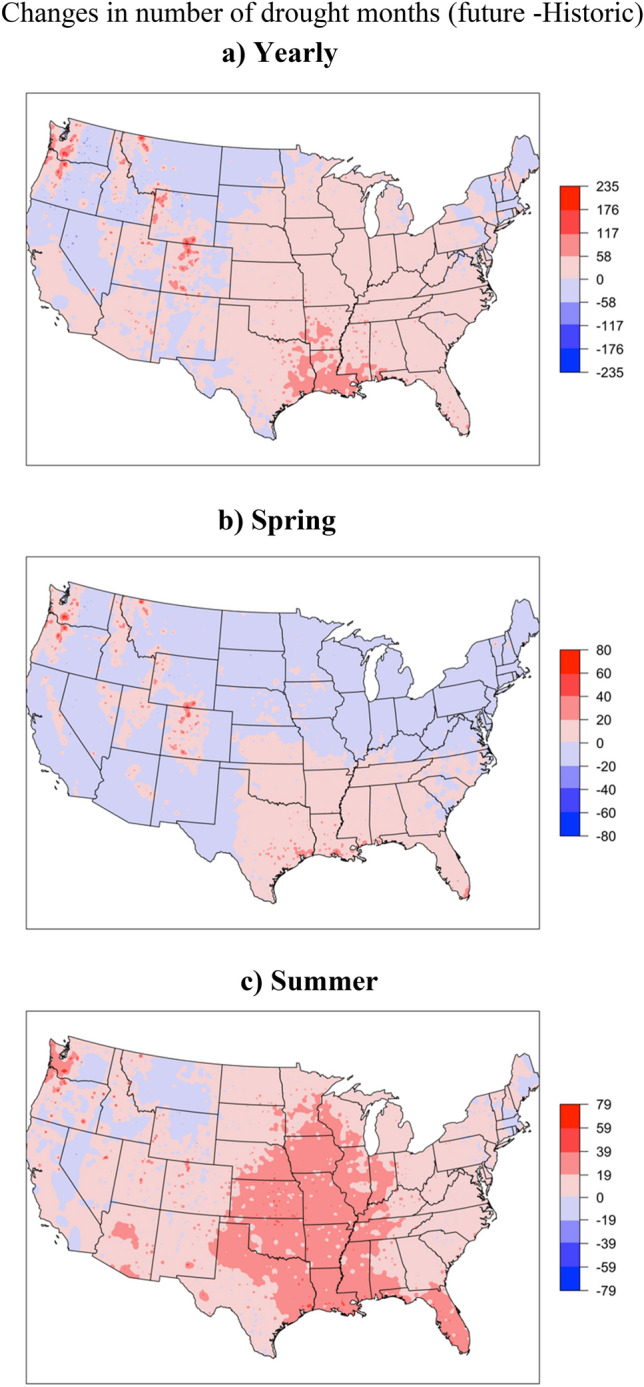
Comparison of change in drought months over 30-year period based on observation (1989–2018) and ensemble mean from the Earth system models future (2023–2052); yearly comparison (**a**), spring comparison (**b**) and summer comparison (**c**).

The comparison of the drought across different CONUS ecoregions showed an increase in drought months in the future (2023–2052) under SSP5 8.5 emission scenarios of CMIP6 compared to the historic (1989–2018) count of drought months except for the Mediterranean California, which showed a decrease in drought months in the future (Fig. 2a,b). The highest increase in extremely dry months (2–3 folds compared to historic) was found in tropical humid forests, temperate sierras and northwestern forested mountain ecoregions (Fig. 2a,b). The North American desert ecoregion showed an increase in the wet months in the future periods compared to historic; extremely wet months increased by around 6 folds. The monthly trends of projected meteorological drought by three ESMs (BCC, CanESM, and UKESM) showed variation in the count and distribution of monthly droughts across different ESMs for different ecoregions (Figure S1). The ensemble approach improved the drought projection by representing a range of future possibilities. Since drought impacts are more important for the crop growing seasons, we compared the seasonal droughts for spring and summer seasons. The results of seasonal drought indicated increase in wet periods in the spring season in the future compared to historic period across majority of ecoregions in CONUS (Fig. 3a,c). In contrast, summer season shows increased drought months for majority of the ecoregions (Fig. 3b,d). Plotting spatially interpolated maps based on the difference in the total count of drought months in ensemble future simulation and observation period to visualize spatial trends of drought across CONUS (Fig. 4a–c) showed increase in the total count of drought months (based in yearly) in future compared to the historic time period. The result showed an increase in drought months in the pacific northwest, central and northern Great Plains (Fig. 4a), eastern Texas, Arkansas, Louisiana and Mississippi (Fig. 4a). For the spring season, majority of the U.S. states showed increases in the wet period except for a few southern states (Fig. 4b). The summer drought month counts showed a significant increase in the eastern temperate forest and great plains regions (Fig. 4c), which includes majority of agricultural land in the CONUS (Fig. 4c). In the Great Plains region, irrigated crop production relies on Ogallala Aquifer, one of the largest freshwater aquifers in the world. Depletion in water levels in Ogallala Aquifer has already affected crop production in the region^51^. Increased drought during summer can add more stress to the limited water available for irrigation, negatively affecting the crop production and the rural economy of the region.

Our results indicate that the drought magnitude will vary across ecoregions. The results based on individual ESMs show some variation in the count and distribution of monthly drought across different ESMs for different ecoregions. However, there was a general agreement among ESM in trends of increased wet months in future spring and drought months in future summer (Figure S2). Previous regional studies conducted in the CONUS showed increased frequency and duration of drought and projected decline in summer precipitation amount under extreme emission scenario of CMIP5 models^17^. These studies were conducted at the site scale, and future projections were determined based on the historical classification of precipitation, streamflow, and soil water content. However, our study provides new insight into the trajectory of drought across the CONUS using multiple models (BCC, CanEMS5, and UKESM) and their ensemble. The overall trends of meteorological drought projections are still incomplete, even though multiple studies have used data from CMIP1 to CMIP5. Our results demonstrated the use of quantile mapping to adjust the CMIP6 precipitation data can improve future representation of temporal and spatial patterns of drought across CONUS. Bias correction helps improve the accuracy and reliability of the model's projections by adjusting the simulated precipitation to match historical observations or other high-quality datasets. Note that the drought projections in this study are based on changes in precipitation alone and do not account for changes in temperature/evapotranspiration/soil moisture. Earlier study using Palmer drought indices (PDI) which uses evapotranspiration and precipitation, suggested an increase in future drought frequency and severity and indicated that the ensemble of multiple models will help better represent drought metric and climate sensitivity^52^. Similarly, the SPI can be easily interpreted and better represents the drought phenomena compared to PDI^53^. The future drought projections show a global increase in potential evapotranspiration driven by elevated temperatures, which leading to increased precipitation extremes, drought frequency and severity across the globe^54–57^.

### Historic and future climate extreme projection for Continental US

**Figure 5:**
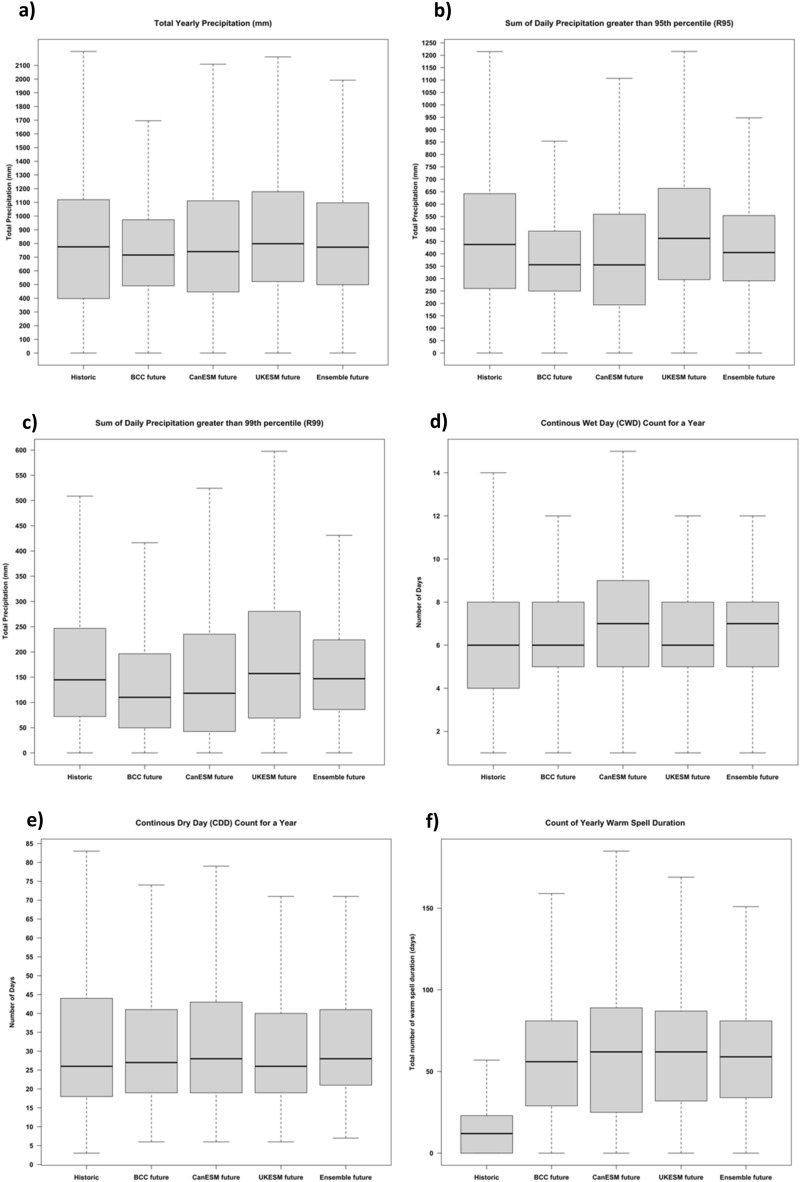
Comparison of yearly count of extreme days for historic based on observed data (1989–2018) and future period (2023–2052) for three Earth system models and its ensemble.

**Figure 6:**
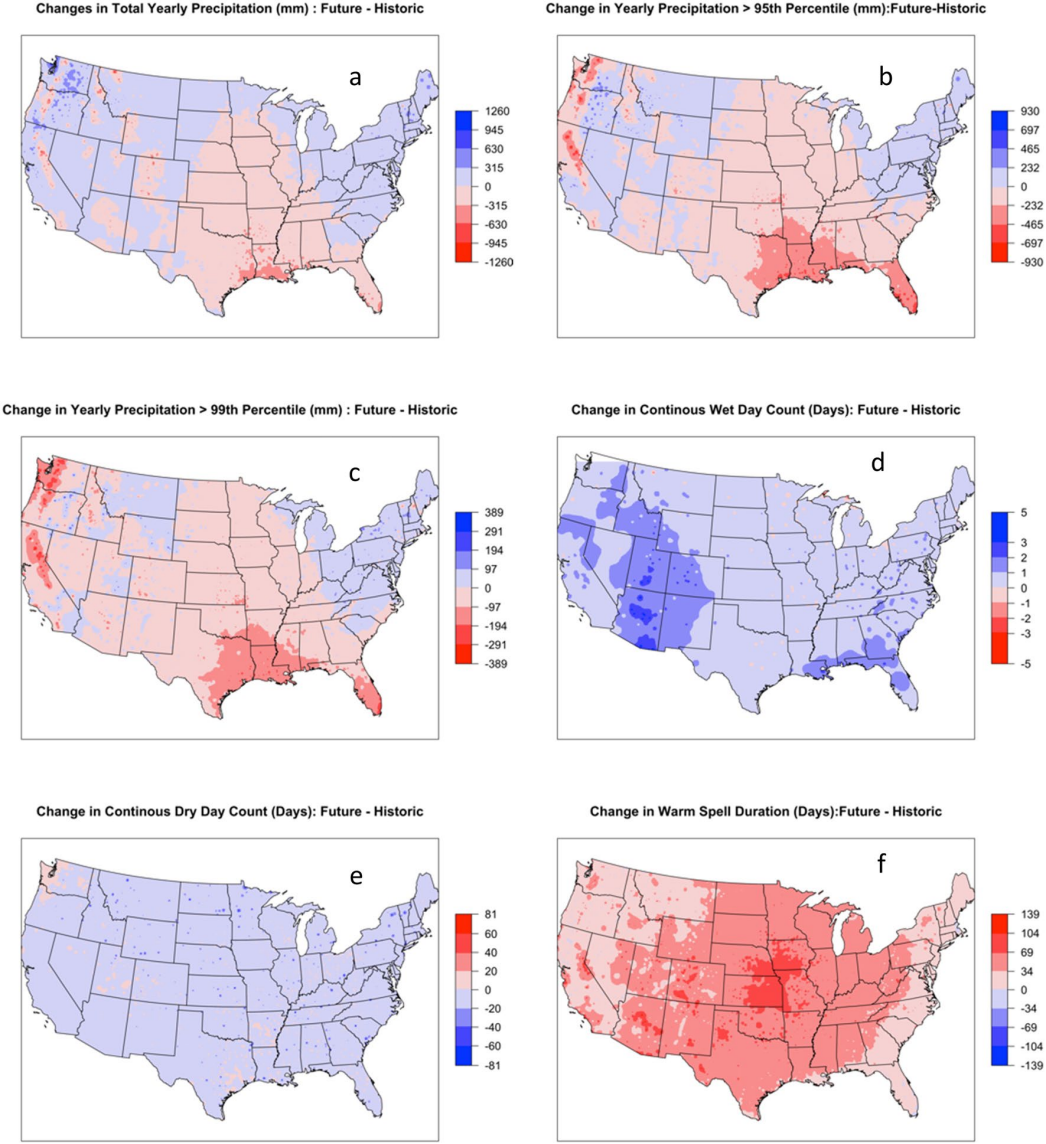
Comparison of change in amount (mm) and count (days) of extreme days for historic (1989–2018) and future period (2023–2052) for three Earth system models and its ensemble.

The projection from different ESMs based on downscaled ESM data shows consensus on increased temperature and precipitation extremes in the future compared to historic observations. The spatial mean annual total precipitation (mm) across CONUS was ${746}_{-255}^{+227}$, ${793}_{-347}^{+319}$ and ${867}_{-345}^{+311}$ in BCC, CanESM and UKESM models, respectively, compared to ${800}_{-400}^{+320}$ mm in observations (Fig. 5a). The overall distribution of total precipitation across CONUS showed decrease in annual precipitation for Eastern temperate forests, Great Plains region, and the state of Texas and Louisiana (Fig. 6a and Figure S3). All three models show an increase in the total precipitation in the western half of the CONUS (Fig. 6a and Figure S3). The detail comparisons of the total yearly precipitation are presented in Figure S3. Previous studies using CMIP5 climate projection have showed an increasing drought trend in the western US^58, 59^. The differences in our results are due to differences in future precipitation projections among CMIP versions and use of different variables for drought calculation. Notably, CMIP6 models indicate increased precipitation for the western USA^60^. An earlier study using uncorrected CMIP6 data reported a higher total annual precipitation, more than 100% in certain ESMs within the western United States^61^. Srivastava et al.^61^ evaluated the daily characteristics of precipitation in historical observations and CMIP6 model historical simulations. They noted an overestimation of wet spell duration across the western US and an underestimation of the dry spell occurrences in the southern US. We demonstrated that the multi-model mean performs better than individual models for capturing precipitation distribution and ensemble analysis helps to get robust inferences by including uncertainty. Results from earlier studies have shown that individual ESM model may be biased and have the potential to either overestimate or underestimate the distribution of precipitation^62^.

The overall distribution of total precipitation (mm) greater than 95th percentile across CONUS showed a decrease in R95 precipitation in great plains and eastern temperate forests (Fig. 6b). The spatial mean annual R95 precipitation (mm) across CONUS were ${386}_{-136}^{+105}$, ${399}_{-205}^{+160}$ and ${506}_{-210}^{+158}$ in BCC, CanESM and UKESM models, respectively, compared to ${470}_{-210}^{+170}$ mm in observations (Fig. 5b). The detail comparisons of the R95 precipitation across different ecoregions are presented in Figure S4. The overall distribution of total precipitation greater than 99th percentile across CONUS showed a decrease in R99 precipitation in great plains and eastern temperate forests (Fig. 5c). The spatial mean annual R99 precipitation across CONUS were ${140}_{-90}^{+56}$, ${159}_{-117}^{+76}$ and ${201}_{-132}^{+79}$ in BCC, CanESM and UKESM models, respectively, compared to ${180}_{-110}^{+70}$ mm in observations (Fig. 5c). The overall distribution of R99 precipitation across CONUS showed decrease in R99 precipitation for Eastern temperate forest and great plains, the trend is similar to total precipitation and R99 (Fig. 6c). The detail comparisons of the R99 precipitation across different ecoregions are presented in Figure S5. The spatial mean annual comparison of consecutive wet days (CWD) across CONUS were ${7}_{-2}^{+1}$, ${8}_{-1}^{+1}$ and ${7}_{-2}^{+1}$ days in BCC, CanESM and UKESM models respectively, compared to ${7}_{-1}^{+2}$ days in observations (Fig. 5d). The overall distribution of CWD across CONUS showed a small increase in wet days across CONUS, with the highest increase in the western United States (Fig. 6d). The detail comparisons of the CWD across different ecoregions are presented in Figure S6. The spatial mean annual comparison of consecutive dry days (CDD) across CONUS were ${37}_{-18}^{+7}$, ${39}_{-20}^{+4}$ and ${36}_{-17}^{+4}$ days in BCC, CanESM and UKESM models respectively compared to ${50}_{-32}^{+6}$ days in observations (Fig. 5e). The overall distribution of CDD across CONUS showed a decrease in dry days across CONUS except for some locations in marine west coast forest (Fig. 6e). The detail comparisons of the CDD across different ecoregions are presented in Figure S7. The overall distribution of WSDI temperature showed an increase in warm spell duration across CONUS. The spatial mean annual WSDI across CONUS were ${56}_{-27}^{+25}$, ${60}_{-35}^{+29}$ and ${60}_{-28}^{+27}$ days in BCC, CanESM and UKESM models respectively compared to ${15}_{-0}^{+8}$ days in observations (Fig. 5f). The overall distribution of WSDI across CONUS shows a significant increase in WSDI in all the ecoregions of CONUS (Fig. 6f). The detail comparisons of the WSDI are presented in Figure S7. Janssen et al.^63^ projected changes in the extreme precipitation trends using the Extreme Precipitation Index (EPI) based on CMIP5 projections for two emission scenarios (RCP 4.5 and 8.5) over the CONUS. The study found an increasing trend of extreme precipitation over the CONUS, mostly an increased in wet periods in spring and dry periods in summer. Both of these seasonal extremes (wet spring and dry summer) will impact the soil water regime and ultimately crop productivity and agricultural sustainability^64, 65^. The Midwest, South Great Plains, Northeast, and Southeast regions all showed an increasing trend in the EPI. The finding from our study based on CMIP6 and earlier study based on CMIP5 showed agreement among model projections of the overall increase in extreme precipitation event frequency in the future. These increases are due to increased atmospheric water vapor and warmer surface temperatures^66^. The study on the projection of temperature and precipitation extremes for CONUS showed a statistically significant increase in extreme events, i.e., decrease in cool nights, and increase in daytime temperature and changes precipitation across regions^67, 68^. Using the generalized extreme value distribution, Lopez‐Cantu et al.^69^ observed increased extreme precipitation magnitude since 1950, and this will continue throughout the twenty-first century in many areas in the US. Kirchmeier-Young and Zhang^70^ studied the extreme precipitation events in North America and found an increasing trend and suggested human emissions further contributed to the increase in these extreme events. The study on temperature extreme based on CMIP6 SSP5 8.5 emission scenario suggested increase in temperature over the northern US of up to 2 °C for 2021–2040, 3 °C 2041–2060 and up to 6 °C 2080–2099 period^71^. This trend was observed mostly in the upper and mid latitude in our study. Given the clear trend of increased precipitation and temperature extremes in future, research should focus on developing adaptation and mitigation scenarios to reduce the impacts of climate extremes. The policy makers should direct their focus to help farmers and stakeholders to adapt more sustainable and regenerative agricultural practices which may be viable to buffer the impacts of future extremes.

Our study has limitation and assumptions. Our study focuses on drought projection using precipitation alone, use of additional variables (plant characteristics, soil moisture and meteorological variables) can help explore other interaction. One of the limitations of quantile mapping includes non-representation of precipitation values above the historic observations.

## Conclusions

Our study used an ensemble of downscaled ESM outputs, drought indices, and extreme indices to assess historic and projected future droughts under a high emission scenario for the CONUS. Findings of our study demonstrated the importance of employing a multi-index approach and utilizing site-specific downscaled climate data for accurate drought projections. When comparing historic observations with climate model outputs, we observed a significant bias in the number of yearly precipitation days and total precipitation amounts. Furthermore, the comparison of drought occurrences across different ecoregions of CONUS revealed an increase in future drought months under the SSP5 8.5 emission scenarios of CMIP6, compared to the historic count of drought months from 1989 to 2018. Most states across CONUS displayed an increase in wet periods, except for a few southern states during the spring season. Notably, there was a notable rise in summer drought months for the Eastern temperate forests and Great Plains regions. The downscaled ESM projections consistently indicated increased temperature and precipitation extremes in the future when compared to historic observations. Across CONUS, the extreme precipitation indices showed a decrease in total precipitation, as well as in the number of very wet days (R95) and extremely wet days (R99) for the Eastern temperate forests and Great Plains regions, which encompass a significant portion of agricultural lands. Particularly, the state of Texas and Louisiana were identified as hotspots for these decreases. Additionally, the distribution of temperature extremes across CONUS exhibited a substantial increase in the WSDI across all ecoregions. These projected changes in climate extremes can have significant implications for agriculture and rural livelihoods. It is imperative for future studies to focus on developing adaptation and mitigation scenarios to minimize the impacts of these climate extremes.

## Materials and methods

### Study area and climate data

**Figure 7:**
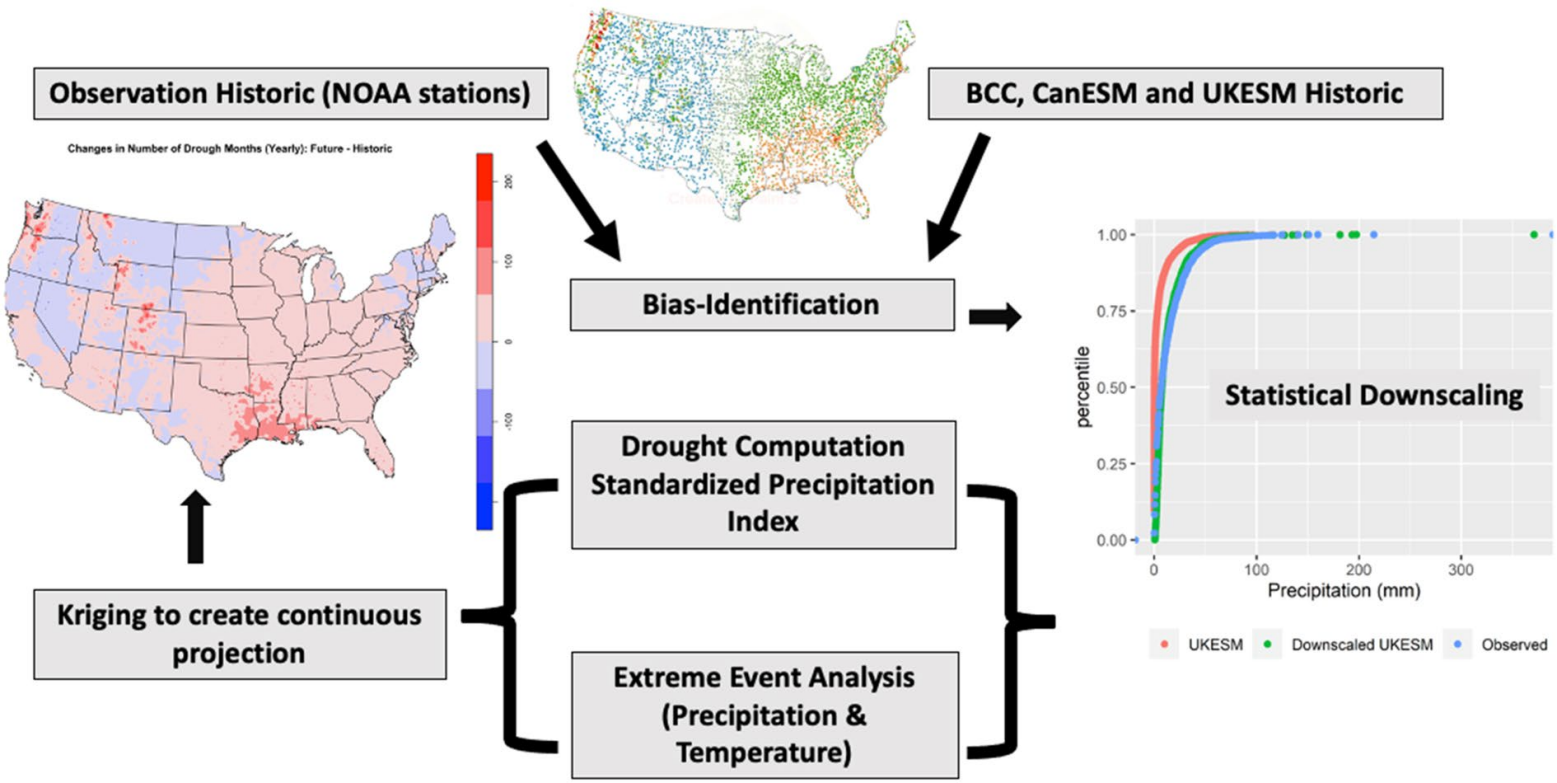
Schematic of approach used in this study for drought and future extreme analysis.

Our study area includes the CONUS which has a large number of National Oceanic and Atmospheric Administration (NOAA) weather stations (Figure S9). The modeling approach used for the calculation of droughts is presented in Fig. 7. There are over 4,161 weather stations with 30-year (1989–2018) datasets across the CONUS. The average annual long-term daily temperature and annual precipitation for the US weather stations is presented in Figure S9 and ecoregion map is presented in Figure S10. The observed historic long-term daily precipitation, and minimum and maximum air temperature data were extracted from the NOAA-Global Historical Climatology Network (GHCN) datasets of National Centers for Environment Information, and used for downscaling and future extreme analysis.

We downloaded and aggregated precipitation, and temperature data from 3 ESM projections for extreme emission scenarios. Data on climate projections used in this study are based on SSP5-8.5, fossil-fueled developed SSP with 8.5 W m^−2^ radiative forcing. The climate models used in this study include Beijing Climate Center Climate System Model (BCC), Canadian Earth System Model (CanEMS5) and UK Earth System Model (UKESM). The selection of ESMs was based on the available spatial variability data range in the CMIP6 deck. We used multiple climate model datasets to represent the range of predictions and uncertainties among CMIP6 models. The SSP5-8.5 scenario was selected to analyze drought occurrences under a projected extreme future emission scenario. SSP5 8.5 represent a high fossil fuel-intensive global trajectory throughout the twenty-first century^72^. The climate model resolutions were 100 km^2^ for BCC, 310 km^2^ for UKESM and 207 km^2^ for CanESM5. Climate data from 1989 to 2018 was used for the historical period, while the future period for analysis was from 2023 to 2052.

### Statistical downscaling of precipitation

Quantile mapping (QM), also known as “probability mapping” and “distribution mapping” were used to adjust the statistical distribution of CMIP6 precipitation data^15, 73, 74^. The quantile mapping corrects the bias in the model projected precipitation values by transforming its statistical distribution to match the observed distribution using a mathematical function. One of the major assumptions of quantile mapping is that the climate distribution doesn’t change over time, i.e., stationarity in the variance and skewness of distribution with only change in mean^75^. For the bias correction, cumulative distribution functions (CDFs) were constructed for both CMIP6 and observed daily historical precipitation data on a monthly basis. The transfer function was used to convert the CMIP6 precipitation values to probabilities based on the model distribution. Finally, their probability was transformed into precipitation values using the quantile function of the observation. The quantile mapping can be mathematically expressed as:

1 $$P_{i,j}^{corr \,esm } = CDF_{obs,j}^{ - 1} \left[ {CDF_{esm,j}^{his} \left( {P_{i,j}^{uncorr \,esm} } \right)} \right]$$

2 $$P_{i,j}^{uncorr \,esm} \left( d \right) = \left\{ {\left. {\begin{array}{*{20}c} {0, if P_{i,j}^{uncorr \,esm} < P_{threshold,j} } \\ {P_{i,j}^{uncorr \,esm} ,otherwise} \\ \end{array} } \right\}} \right.$$

where ${P}_{i,j}^{uncorr \,esm}$, ${P}_{i,j}^{corr \,esm}$ and ${P}_{max,j}^{cal \,esm}$ are the projected uncorrected CMIP6 and corrected CMIP6, respectively. The ${CDF}_{obs,j}^{-1}$ is the inverse CDF of the observed precipitation and ${CDF}_{esm,j}^{hist}$ is the CDF of an individual ESM. These values were computed using historical observed precipitation and ESM simulated historical precipitation data.

Quantile mapping was modified to address a major limitation in CMIP6 P data: the P frequency. The CMIP6 model outputs exhibits a relatively high precipitation frequency or dry bias. This dry bias is characterized by a large number of very low P days, often referred to as “drizzle days” that occur in CMIP6 model outputs due to spatial aggregation. To correct this bias for each model, we compared the historical observed dataset with historical ESM data. We identified a monthly threshold for the ESM data, and values smaller than this threshold were excluded to align the number of ESM precipitation days with historical observations. However, it’s important to note that our approach has limitations, including the inability to accurately represent precipitation values exceeding those observed in historic observations. SPI incorporates the changes in frequency of precipitation events and shift in precipitation pattern in the future which are critical for the projection of drought. For each of the climate model grid nearest weather station from the centroid of the grid was assigned based on Euclidean distance.

### Precipitation and temperature based extreme indices

The drought indices and extreme values were calculated on a monthly basis to estimate the future trajectory of drought and extreme conditions over CONUS. The drought index was calculated using the observed historic data sets and future drought was quantified based on the threshold precipitation values identified in the historic drought estimation. The extreme indices were used to characterize the temperature and precipitation related extremes, e.g., prolonged dryness, wetness, coldness and hotness (Table 1). Monthly drought and extreme indices were calculated using daily precipitation and temperature data. The projections were made for CONUS ecoregions over a 30-year observed period (1989–2018) and a 30-year projected period (2023–2052). Moreover, temperature and precipitation trends were also used to investigate climate extremes. Note all the drought and extreme calculation were made at point scale (for each weather stations) and the spatial map presented in the result are geographically weighted kriging of those prediction.

**Table 1:** List of extreme temperature and precipitation indices used in this study.

Short name	Indicator	Definition	Unit
CDD	Consecutive dry days	Maximum number of consecutive days without PRCP	days
CWD	Consecutive wet days	Maximum number of consecutive days with P	days
R95	Very wet days	Annual total PRCP when RR > 95th percent	mm
R99	Extremely wet days	Annual total PRCP when RR > 99th percent	mm
PRCPTOT	Annual total wet-day precipitation	Annual total PRCP on wet d	mm
WDSI	Warm spell duration indicator	Annual count of days with at least 6 consecutive days when TX > 90th percent	days

### Standardized precipitation index

The meteorological drought was computed using the precipitation data using well established standardized precipitation index (SPI)^76, 77^. The SPI gives the relative measure of the dryness and wetness based on the long-term precipitation records. Precipitation generally follows gamma distribution as its empirical distribution tends to have positive skewness. The SPI calculation is done on a monthly basis, where the precipitation data for each of the 12 months are fitted to a two-parameter gamma distribution. The gamma distribution is fitted using maximum likelihood estimation of its parameters α and β (Eq. 3). The inverse function is then applied to the cumulative probability to calculate the SPI values for each month (Eq. 4). The standardized indices for the future were counted based on the threshold range of precipitation values for each of the drought class for different months. As the SPI calculation are normalized to the time period, we used the threshold value from the historical calculation for the estimation of future drought. Positive SPI values imply higher median precipitation or wet conditions, while negative values suggest dry conditions; thus, monthly SPI is critical for understanding the association of soil moisture and crop stress during the growing season since it is a more short-term value. The SPI values and respective drought class is presented in supplement table The SPI was calculated using the following equations:

3 $$g\left( x \right) = \frac{1}{{\beta^{\alpha } \Gamma \left( \alpha \right)}}x^{\alpha - 1} e^{{\frac{ - x}{\beta }}} ,\quad for\;\;x > 0$$

4 $$\Gamma \left( \alpha \right) = \mathop \smallint \limits_{0}^{\infty } x^{\alpha - 1} e^{ - x} dx$$

where x is the monthly precipitation, $\Gamma \left(\alpha \right)$ is the gamma function, and g(x) is the probability density function of the gamma distribution. The shape and scale parameters α and β are estimated by the maximum likelihood method as shown in Eqs. (5) and (6).

5 $$\alpha = \frac{1}{4A}\left( {1 + \sqrt {1 + \frac{4A}{3}} } \right), \;\;\beta = \frac{{\overline{x}}}{\alpha }$$

6 $$A = ln\left( {\overline{x}} \right) - \frac{\sum ln\left( x \right)}{n}$$

where *A*, n, $\overline{x }$ are precipitation factors for calculating shape parameters, number of months and mean precipitation. The resulting parameters are then used to find the cumulative probability of precipitation for the given month as follows:

7 $$G\left( X \right) = \frac{1}{{\beta^{\alpha } \Gamma \left( \alpha \right)}}\mathop \smallint \limits_{0}^{x} x^{\alpha } e^{{\frac{ - x}{\beta }}} dx$$

The cumulative probability G(X) is transformed to standard normal random variable z with a mean of zero and variance of one, which is named SPI.

### Extreme indices of precipitation and temperature

The trend and statistics of the precipitation and temperature values can be used to explore the climate extremes. The extreme calculation using multiple variables can give meaningful conclusion on actual impact assessment. The extreme indices were calculated using the downscaled climate datasets. In this study, we standardized indices from Expert Team on Climate Change Detection and Indices (ETCCDI) using the daily precipitation and temperature data^78^. Similar to the future drought calculation, the threshold value from the historical data was used to compute future extreme for very wet days (R95p) and extremely wet days (R99p), and warm spell duration indicator (WSDI). The consecutive dry days (CDD), consecutive wet days (CWD), and total precipitation (PRCPTOT) calculations were done independently for the historic and future periods (Table 1).

### Supplementary Information

Supplementary Information.

## Data Availability

All the data that support the finding of the study is presented in the paper, including Figure and supplement materials and data supporting the findings of this study are available on DRYAD [https://doi.org/10.5061/dryad.bzkh189g].
