# Supplementary material for: Increased drought and extreme events over continental United States under high emissions scenario
Source: Sci Rep. 2023 Dec 6;13:21503. doi: 10.1038/s41598-023-48650-z (PMC10700340; doi:10.1038/s41598-023-48650-z)
Supplement: Supplementary file 1 — Supplementary Information. [file 41598_2023_48650_MOESM1_ESM.docx]

**Supplementary Information**

**Increased drought and extreme events over continental United States under high emissions scenario**

Sagar Gautam*^1,2^, Umakant Mishra^1,2^, Corinne D Scown^2,3,4,5^ & Rajan Ghimire^6^

^1^ Bioscience Division, Sandia National Laboratory, Livermore, CA 94550, United States

^2^ Joint BioEnergy Institute, Lawrence Berkeley National Laboratory, Emeryville, CA 94608, United States.

^3^Energy Analysis & Environmental Impact Division, Lawrence Berkeley National Laboratory, Berkeley, CA 94720, United States.

^4^ Biological Systems and Engineering Division, Lawrence Berkeley National Laboratory, Berkeley, CA 94720, United States.

^5^ Energy & Biosciences Institute, University of California, Berkeley, CA 94720, United States.

^6^Agricultural Science Center, New Mexico State University, NM 88101, USA

E-mail: sgautam@sandia.gov


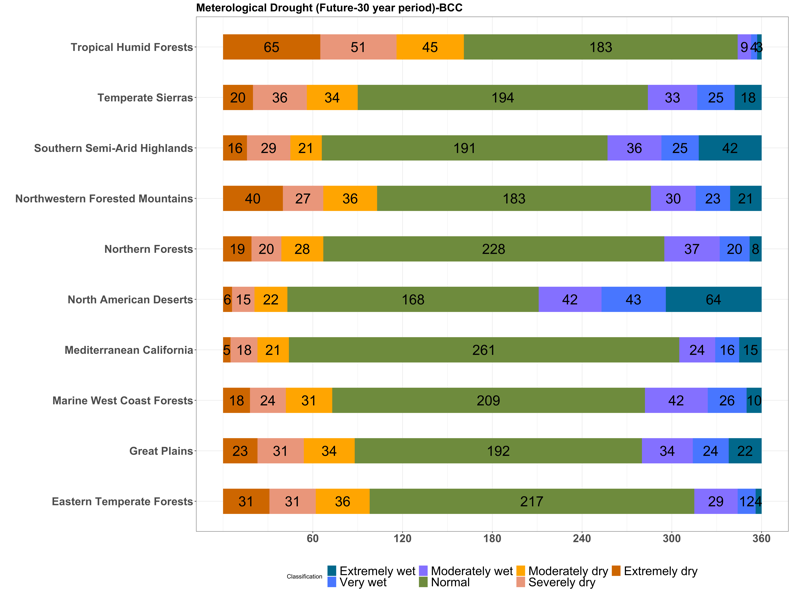


**a)**


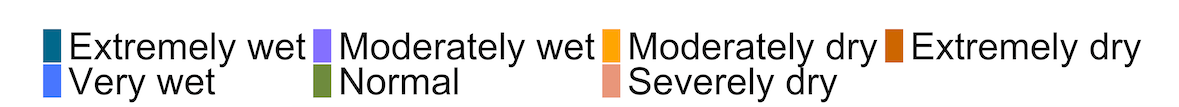


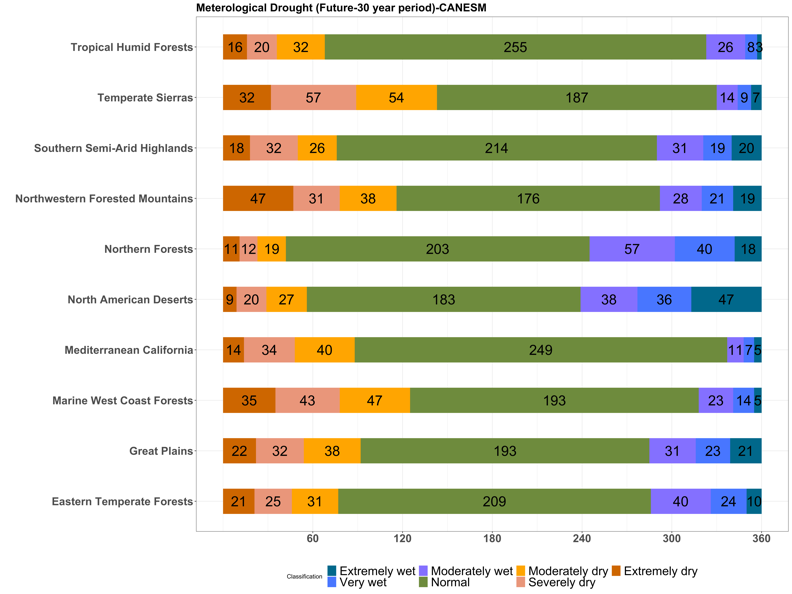


**b)**


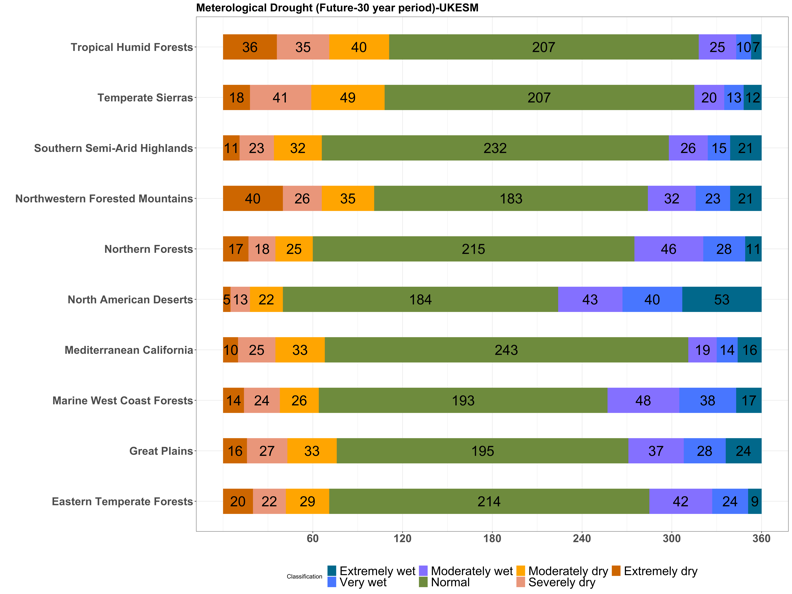


**c****)**

**Figure S1.** Comparison of meteorological drought across different U.S. ecoregions during 30-year (2023-2054) future period for three ESMs; BCC (a), CanESM (b) and UKESM (c).

**c)**

**b)**

**a)**


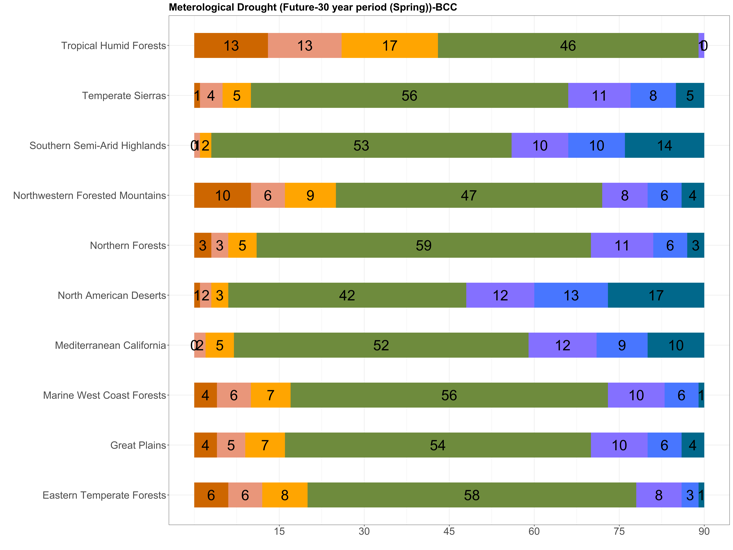

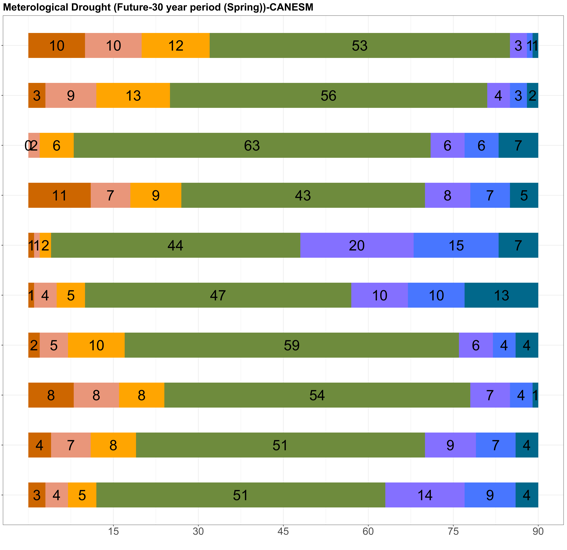

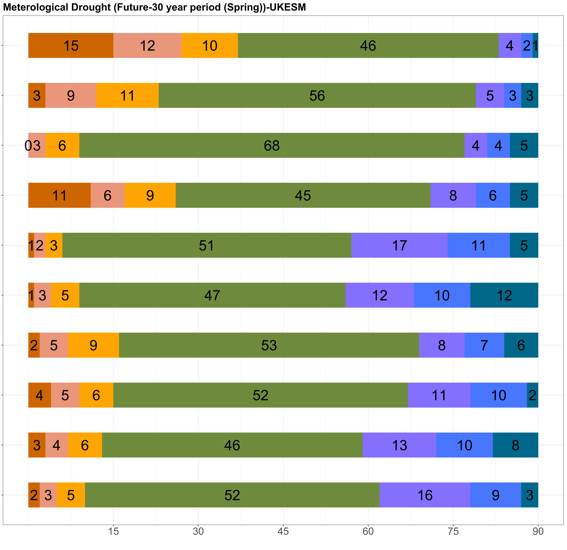


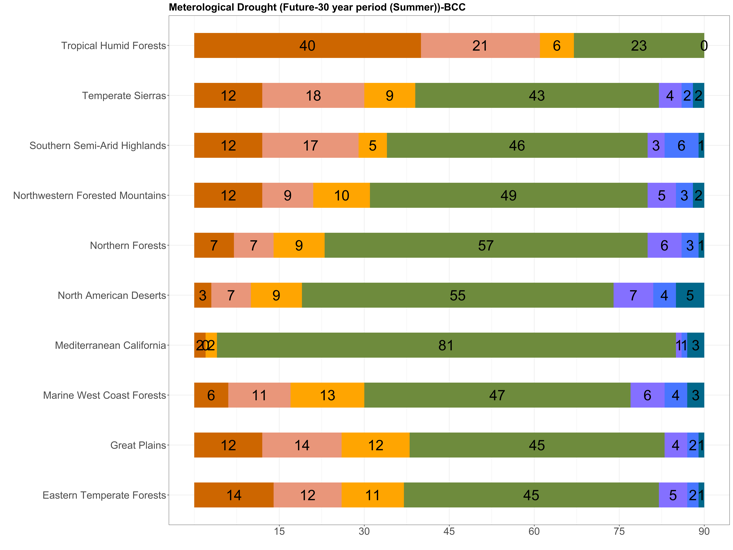

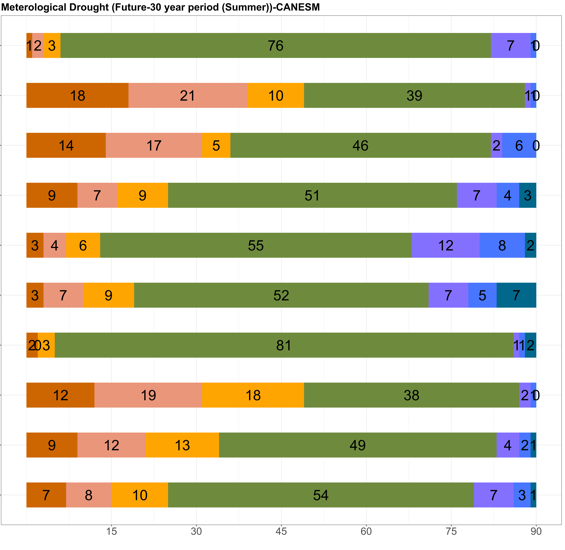

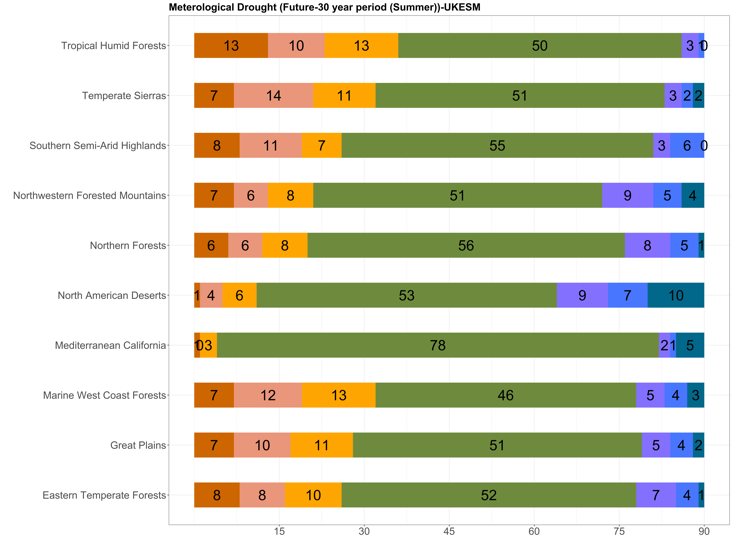


**e)**

**d)**

**f)**


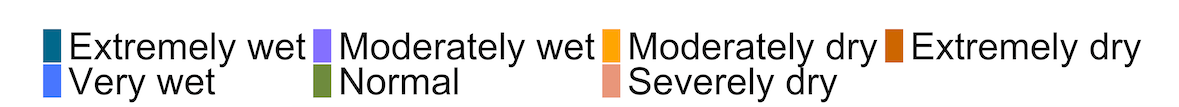


**Figure S2.** Comparison of seasonal meteorological drought across different ecoregions for 30-year (2023-2054) future period. Upper three figure for historic spring BCC (a) CanESM (b) and UKESM (c) and lower three figure for future spring BCC (d) CanESM (e) and UKESM (f).

**
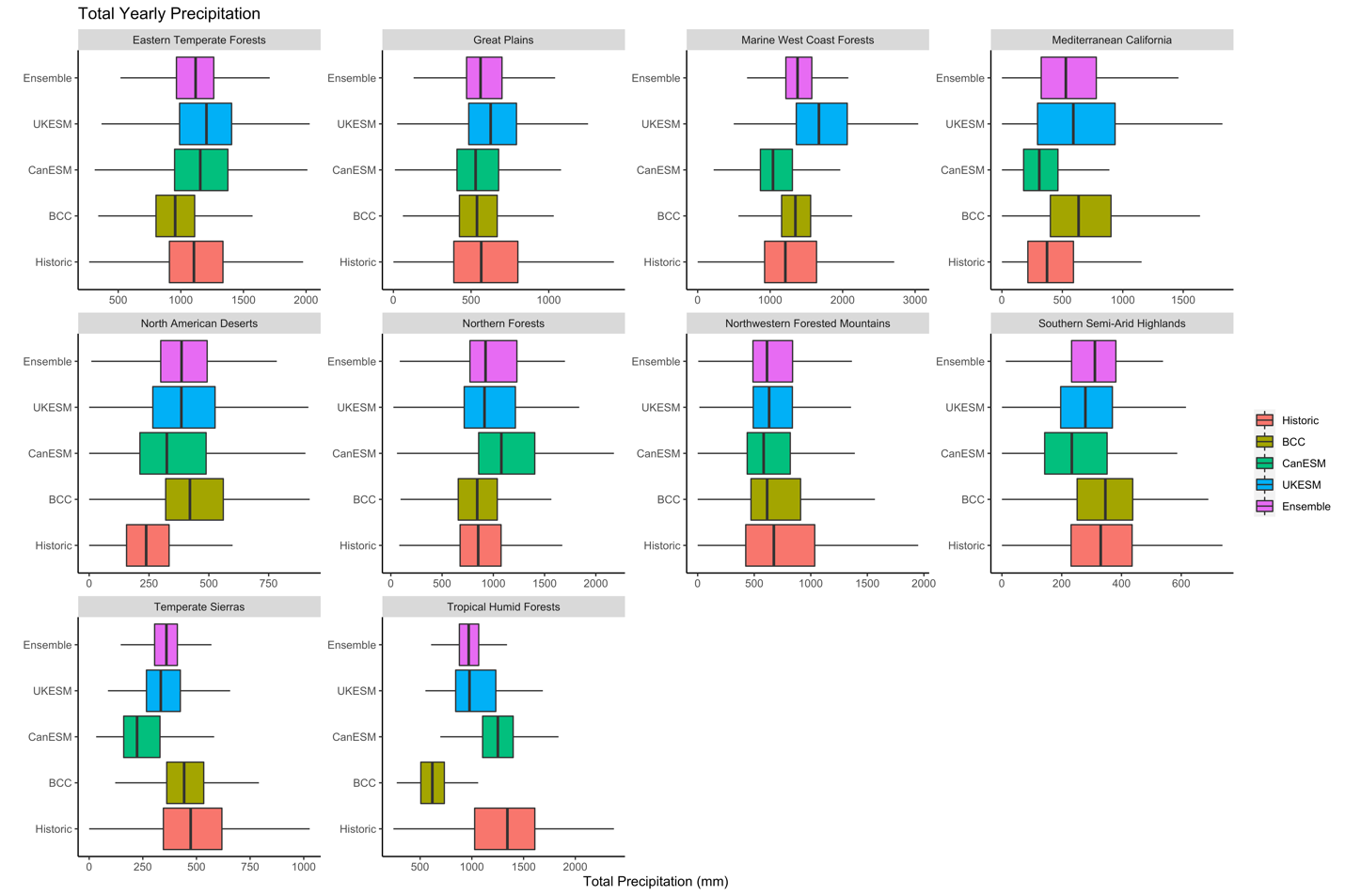
**

**Figure S3.** Comparison of total yearly precipitation for historic and future period for three Earth system models and its ensemble across different ecoregion.


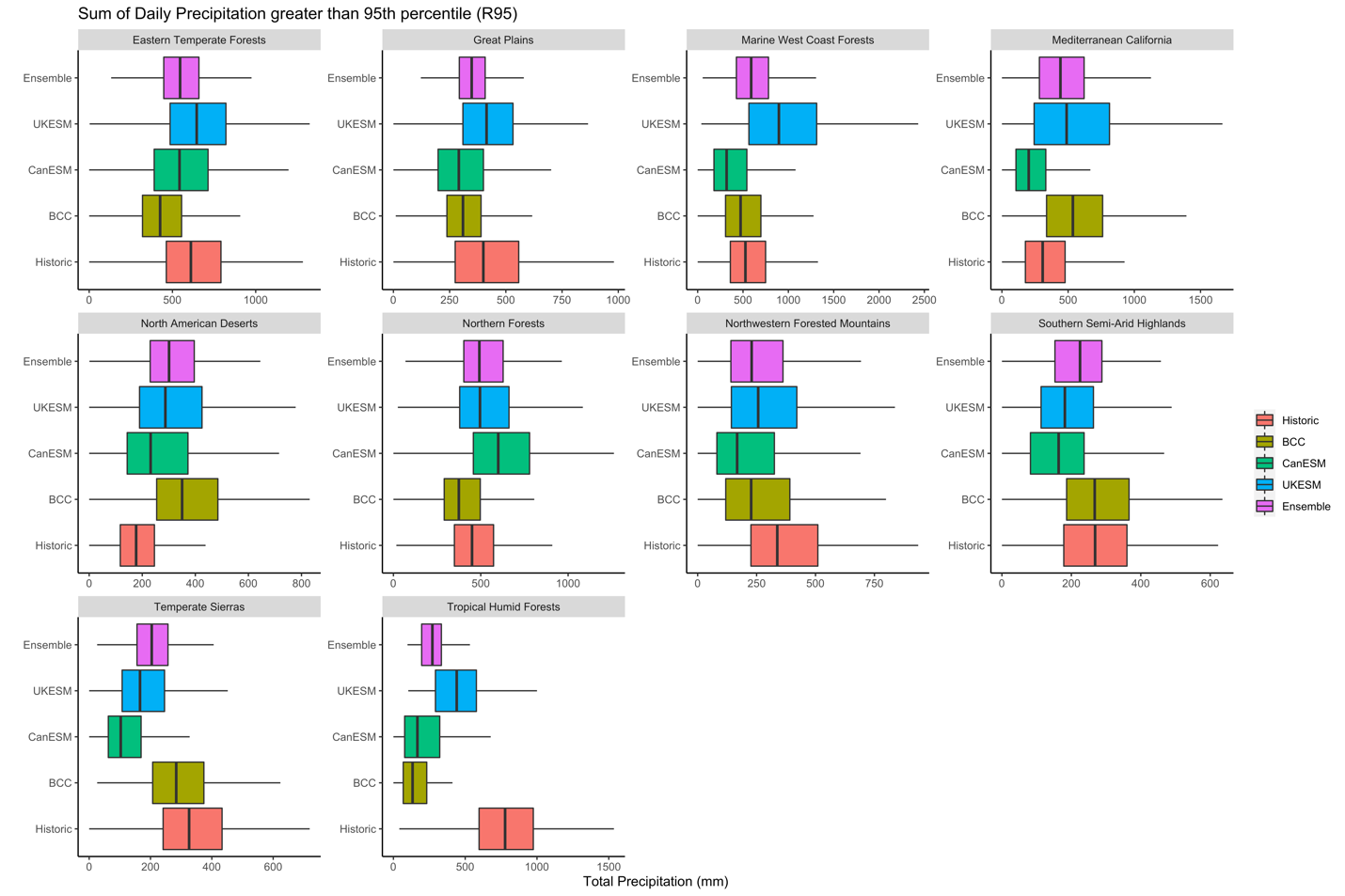


**Figure S4.** Comparison of total yearly precipitation greater than 95^th^ percentile (R95) for historic and future period for three Earth system models and its ensemble across different ecoregion.

**
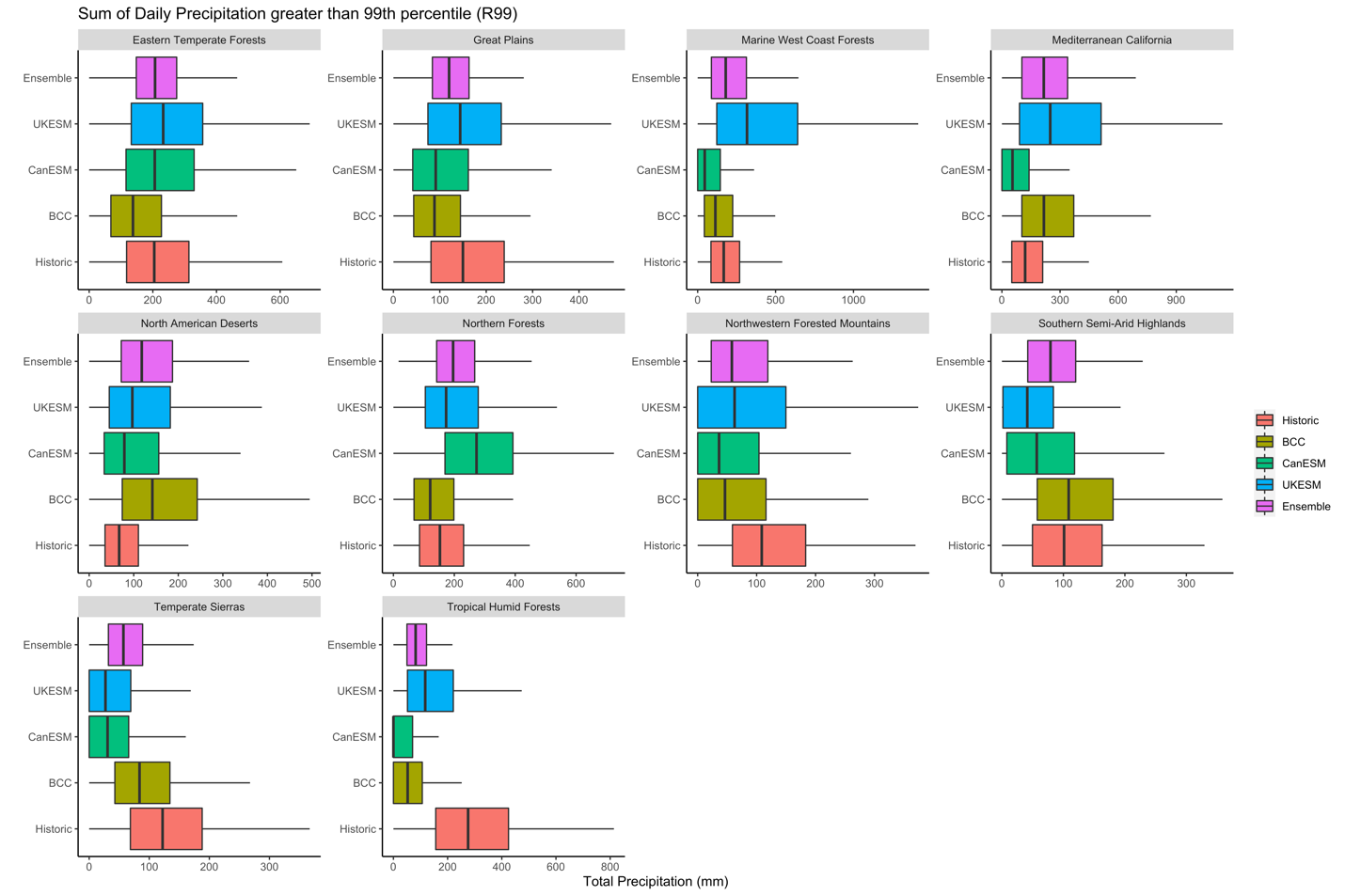
**

**Figure S5.** Comparison of total yearly precipitation greater than 99^th^ percentile (R99) for historic and future period for three Earth system models and its ensemble across different ecoregion.


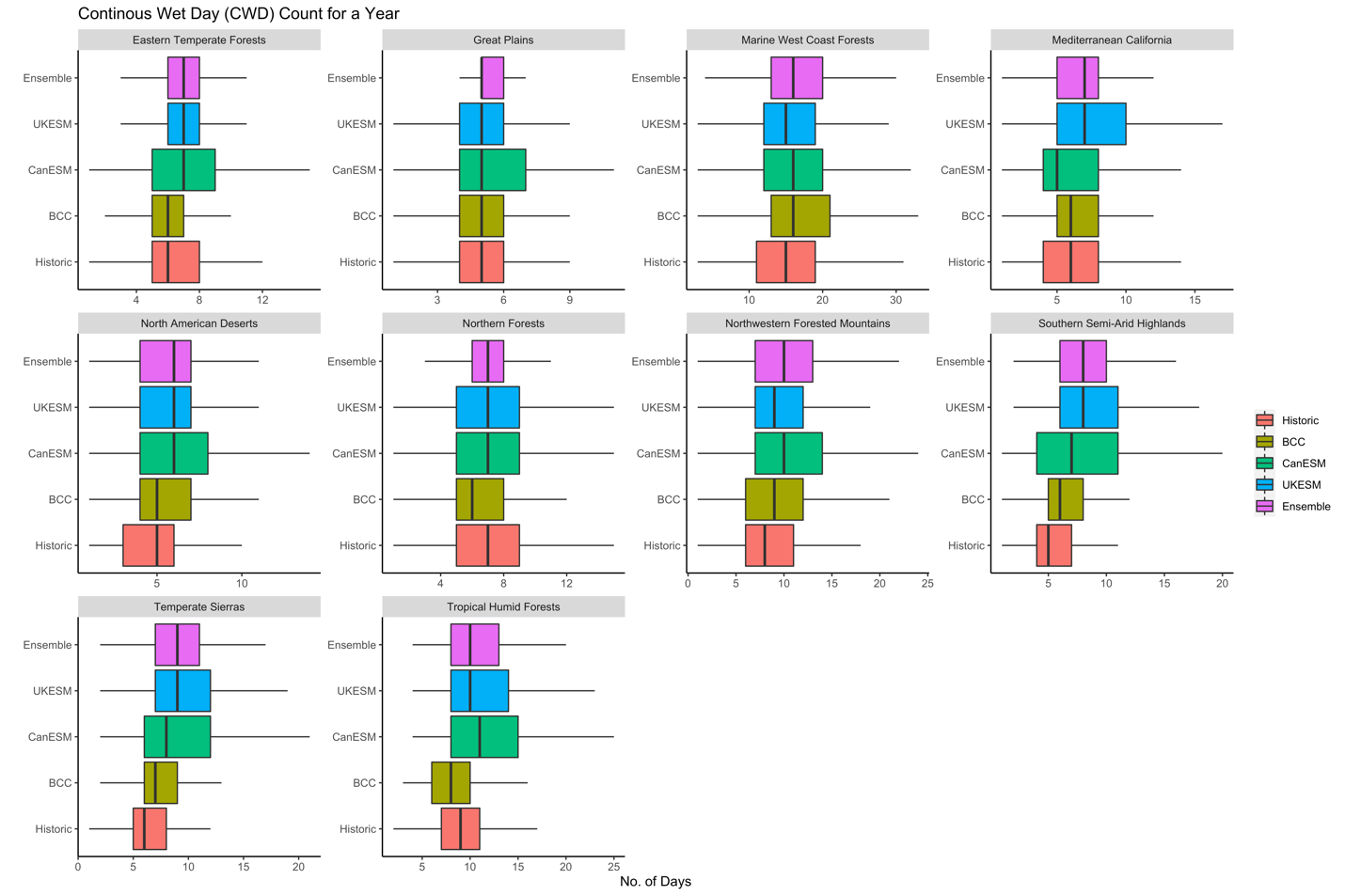


**Figure S6.** Comparison of yearly count of continuous wet days (CWD) for historic and future period for three Earth system models and its ensemble across different ecoregion.


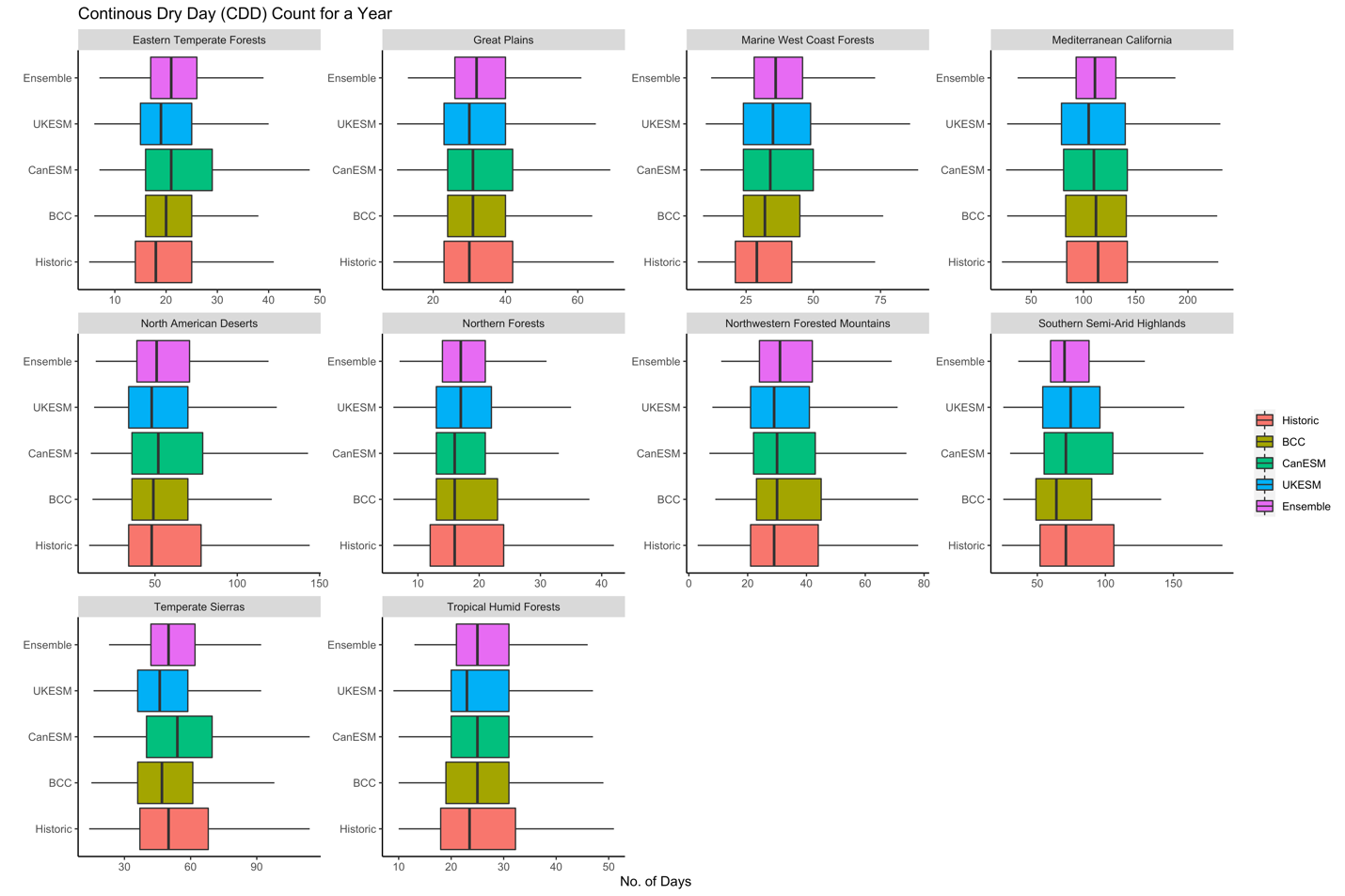


**Figure S7.** Comparison of yearly count of continuous dry days (CDD) for historic and future period for three Earth system models and its ensemble across different ecoregion.


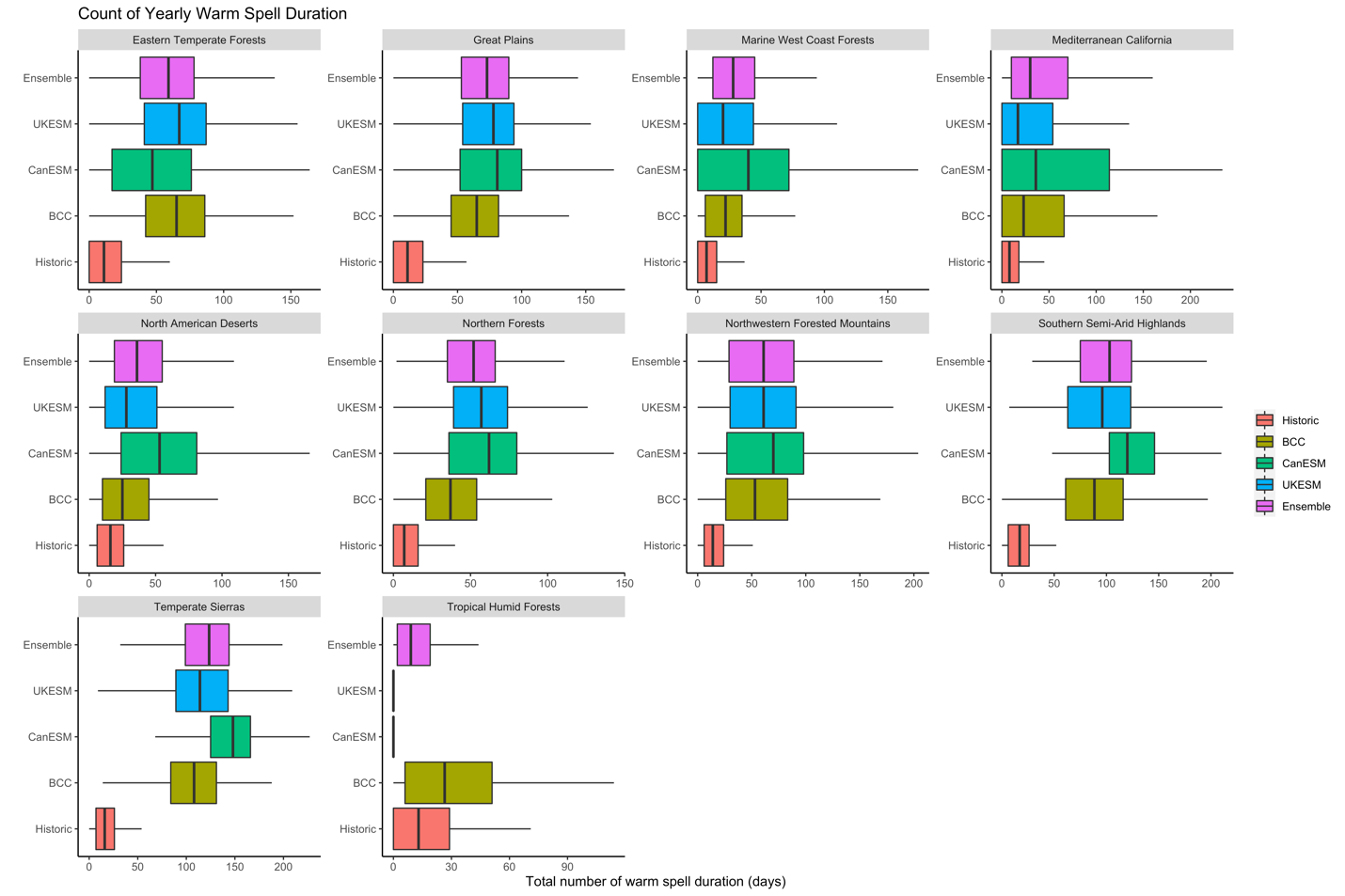


**Figure S8.** Comparison of yearly count warm spell duration days for historic and future period for three Earth system models and its ensemble across different ecoregion.


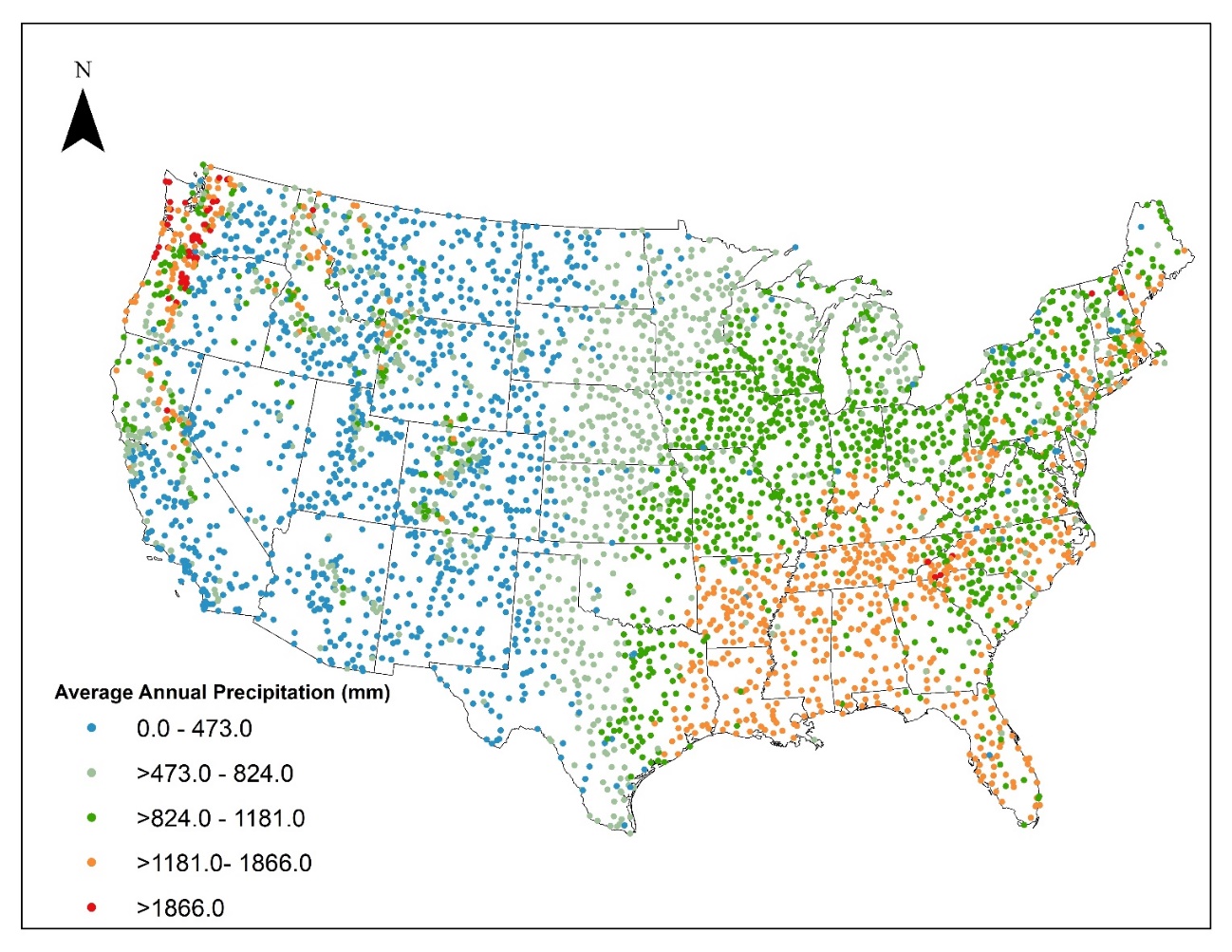


**Figure S9.** Spatial distribution of 4,161 weather stations with long-term climate observations across continental U.S.


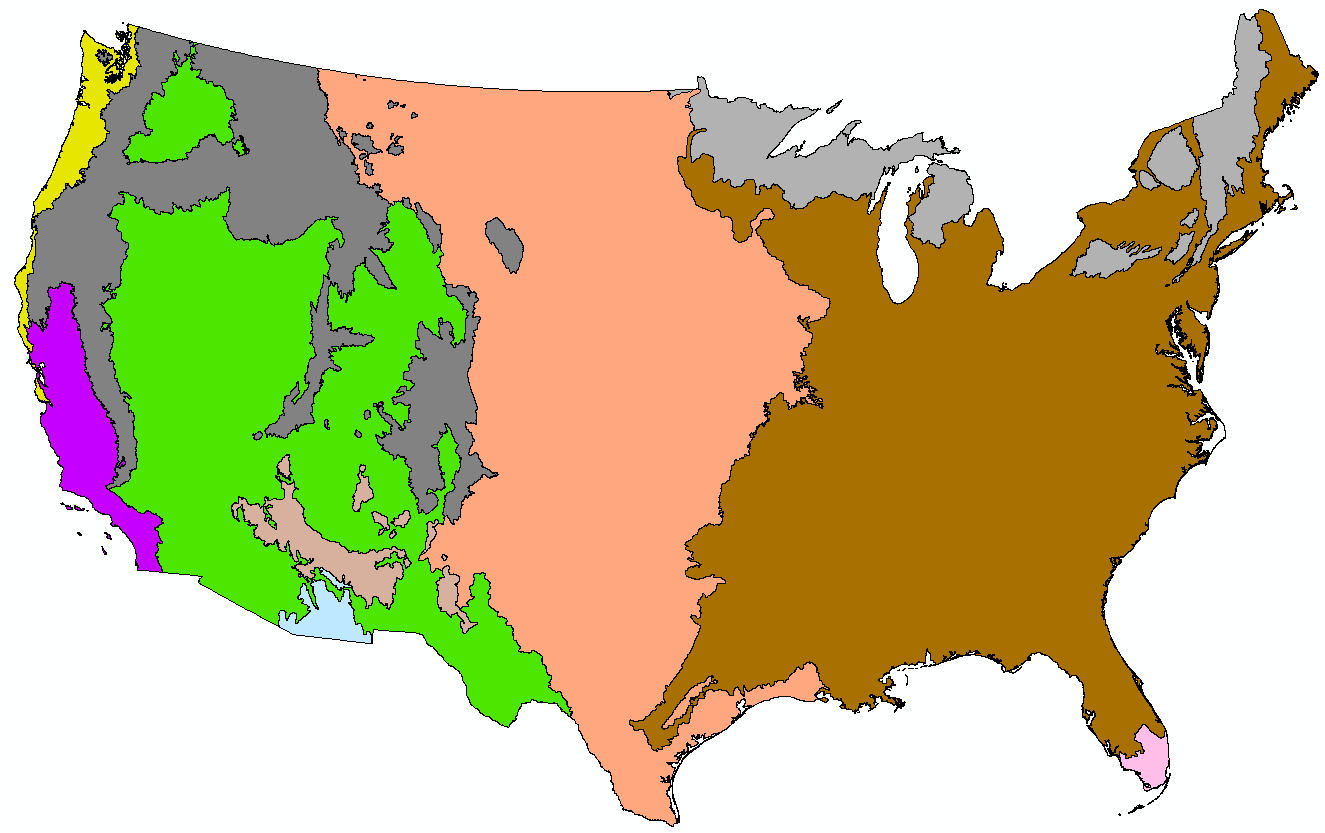

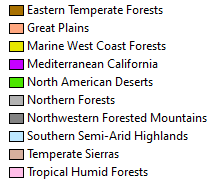


**Figure S10.** Spatial distribution of continental U.S. ecoregions used in this study.

Table S1. Drought index class definition for standardized precipitation index (adapted from Zhang, et al. ^1^)

| standardized precipitation index | Classification |
| --- | --- |
| 2.0 > | Extremely wet |
| 1.5 to 1.99 | Very wet |
| 1.0 to 1.49 | Moderately Wet |
| -0.99 to 0.99 | Normal |
| -1 to -1.49 | Moderately Dry |
| -1.5 to -1.99 | Severely Dry |
| -2.0 < | Extremely Dry |

Reference

1 Zhang, X. *et al.* Indices for monitoring changes in extremes based on daily temperature and precipitation data. *Wiley Interdisciplinary Reviews: Climate Change* **2**, 851-870 (2011).
